# Dual-View Complementary Multiscale Entropy Fusion for Rotating-Machinery Fault Diagnosis

**DOI:** 10.3390/e28101150

**Published:** 2026-10-20

**Authors:** Wei Xiao, Yang Zhang, Rui Xu

**Affiliations:** 1 Business School, Hohai University, Nanjing 211100, China; wei.xiao@hhu.edu.cn; 2 School of Automation, Xi’an University of Posts and Telecommunications, Xi’an 710121, China; zhangyang06@stu.xupt.edu.cn

**Keywords:** rotating machinery, fault diagnosis, entropy features, multiscale entropy, vibration analysis, feature fusion, grouped cross-validation

## Abstract

Entropy-based fault diagnosis depends on signal representation and the properties captured by each entropy measure. We evaluated dual-view complementary multiscale entropy fusion (DCMEF), which combines RMS-normalized raw vibration with a normalized logarithmic Hilbert envelope. Three entropy measures at five fixed scales produce 30 candidate features, with imputation, selection, and classifier tuning confined to grouped training folds. In an exploratory comparison against 20 representations across four selected tasks and three classifiers, DCMEF achieved the highest observed accuracy in nine of 12 settings and tied for highest in three. A separate view ablation found that the full bank outperformed raw-only features in two settings, tied in five, and underperformed in five; relative to envelope-only, it outperformed in ten and tied in two. Thus, raw-vibration features were useful across these settings, whereas adding envelope features had inconsistent effects. Independent evaluation is needed to assess performance beyond the selected tasks.

## 1. Introduction

Gears and rolling bearings are critical components in rotating machinery, and their operating condition has direct implications for production continuity, equipment safety, and maintenance costs. Vibration-based fault diagnosis is widely employed for condition monitoring because mechanical defects induce characteristic changes in the dynamic response of rotating systems. In practical applications, however, fault-related signatures are often superimposed on variations in operating conditions, background vibration, transient disturbances, and speed- or load-dependent modulation. Consequently, extracting informative and discriminative features from such non-stationary vibration signals remains a fundamental problem in rotating-machinery condition monitoring [1,2].

Entropy-based features provide compact measures of temporal structure, irregularity, and signal complexity, while different entropy families characterize distinct properties of the underlying dynamics. Permutation entropy (PE) quantifies local ordinal patterns [3]; sample entropy and fuzzy entropy assess the recurrence and similarity of embedded templates [4,5]; fluctuation-based dispersion entropy characterizes transitions between amplitude-derived symbolic classes [6,7]; and diversity entropy quantifies the diversity of state distributions [8]. Although these measures are related, they capture different aspects of signal dynamics and are therefore not interchangeable. A representation based on a single entropy family may characterize only part of the information contained in vibration signals, while other dynamical properties remain insufficiently represented.

The multiscale framework extends entropy analysis to multiple temporal resolutions, allowing signal characteristics to be examined across different scales [9,10]. Composite multiscale methods construct multiple offset coarse-grained sequences to reduce estimation variability [11], whereas refined composite methods aggregate probability or similarity statistics across offsets before computing the final entropy value [12]. In rotating-machinery fault diagnosis, multiscale permutation entropy [13], composite weighted permutation entropy [14], envelope-based multiscale entropy [15], composite multivariate multiscale permutation entropy [16], and refined composite generalized multiscale dispersion entropy [17] have been applied under different datasets and classification settings. Their diagnostic performance, however, varies with the entropy formulation, multiscale construction, signal representation, and evaluation protocol. A recent benchmark study also reported marked differences among multiscale entropy methods in both diagnostic accuracy and computational cost [18]. These results indicate that no single entropy measure is uniformly preferable across diagnostic settings and provide a basis for combining complementary entropy mechanisms within a unified representation.

Recent studies have pursued related diagnostic questions with learned representations. Cross-sensor self-contrastive learning and multi-source fusion address limited labels or complementary sensor streams [19,20]. Interpretable subdomain adaptation networks address distribution shifts between bearing operating conditions, including designs that incorporate physical information into the feature mapping [21,22]. Few-shot continual diagnosis, variational fusion, and vibration–text alignment address other forms of adaptation and representation learning [23,24,25], while graph distillation and physics-informed hypergraph models have been used for bearing remaining-useful-life prediction [26,27]. These studies differ from the present single-channel entropy-feature comparison in their inputs, learning setting, or prediction target; their published accuracies are not directly comparable with the results reported here.

Signal representation is another design choice in entropy-based fault diagnosis. The normalized raw waveform retains broadband oscillatory information, whereas the analytic-signal envelope emphasizes amplitude modulation. Envelope-based multiscale analysis has been applied to bearing diagnosis under variable speed [15], but replacing the raw waveform with its envelope can also discard useful structure. DCMEF retains both views as candidate descriptions rather than assuming that either view always improves accuracy. Because concatenating entropy features may introduce near-constant or redundant variables, feature control is fitted within each training fold.

To examine the diagnostic value of combining these signal views, dual-view complementary multiscale entropy fusion (DCMEF) was developed as a feature framework rather than as a new elementary entropy definition. For each vibration window, DCMEF constructs an RMS-normalized raw-vibration view and a normalized logarithmic Hilbert-envelope view. Multiscale permutation entropy (MPE), multiscale fuzzy entropy (MFE), and refined composite multiscale fluctuation-based dispersion entropy (RCMFDE) are computed at scales 1, 2, 3, 5, and 8, yielding 30 candidate features that characterize ordinal dynamics, fuzzy local similarity, and amplitude-level fluctuations. Within each outer training fold, the pipeline imputes undefined values, removes near-constant and highly correlated features, ranks the remaining features using the Fisher score, selects the number of retained features through grouped inner cross-validation, and standardizes the selected feature subset.

We compared DCMEF with 20 single-scale, multiscale, composite, and refined composite entropy representations using Extra Trees, Random Forest, and K-nearest neighbors. The four selected group-held-out tasks cover SEU and HUST gearboxes and UOttawa and HUST bearings. This study contributes a defined dual-view candidate feature bank and a training-fold-only feature-control pipeline and then examines their performance within each task and classifier. A separate view ablation tests whether the full bank improves on each single-view bank. Because the unified task and classifier set was fixed after earlier results had been inspected, these comparisons are exploratory.

## 2. Method

### 2.1. Overview and Notation

DCMEF converts a vibration record into a diagnosis through five sequential stages: deterministic windowing, dual-view construction, complementary multiscale entropy extraction, training-fold-only feature control, and decision aggregation. Let a vibration record be

*x* = {*x*_1_, *x*_2_, …, *x_N_*} (1)

Complete windows of length *L* = 2048 are extracted with a step size of *H* = 1024 samples:

*x*^(*q*)^ = {*x*_1+(*q*−1)*H*_, …, *x_L_*_+(*q*−1)*H*_} (2)

No zero padding is applied. If the number of admissible windows exceeds the dataset-specific maximum, the retained windows are selected deterministically from the valid starting positions. Each window inherits the identifier, class label, and outer-validation group of its source record or block. Group membership is preserved during model evaluation, ensuring that no window from a held-out group is included in the corresponding training fold.

For each window, DCMEF constructs two signal views and computes three entropy profiles at five scales. The fixed scale set is

(3) S={1,2,3,5,8}

**Figure 1:**
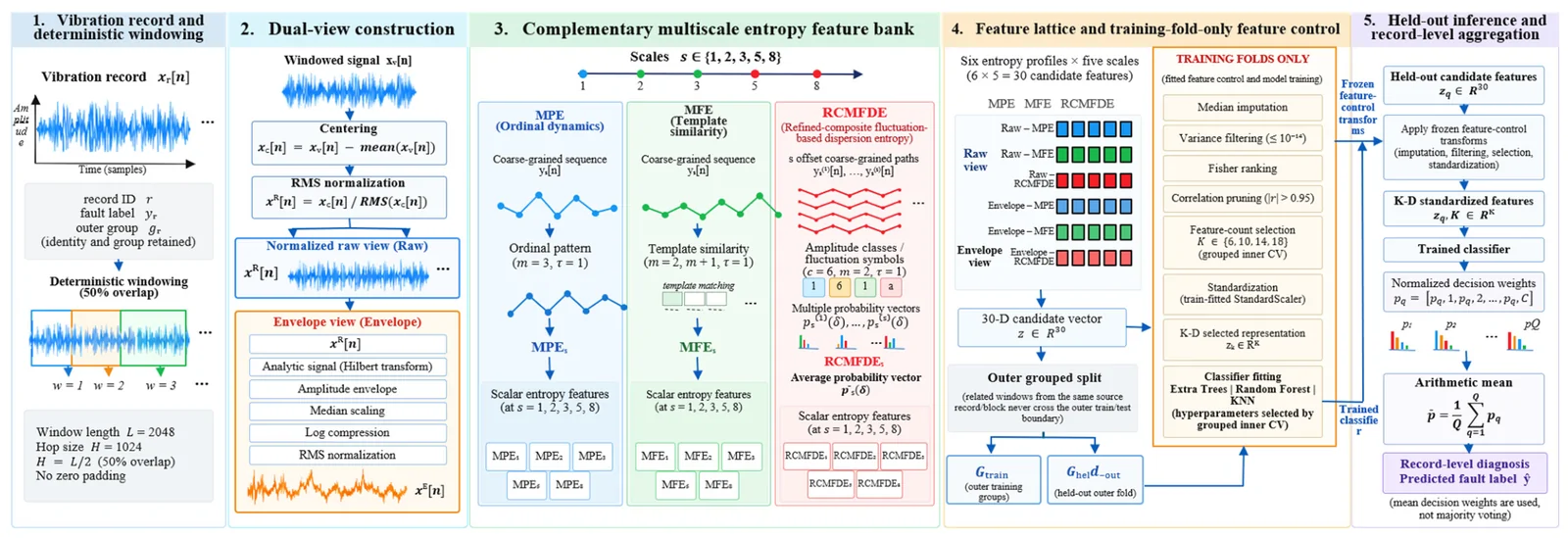
Dual-view complementary multiscale entropy fusion (DCMEF). Blue, green, and red denote MPE, MFE, and RCMFDE; orange denotes the envelope view. Ellipses indicate repeated windows or computational steps.

The six combinations of two signal views and three entropy measures each yield five feature values, forming a 30-dimensional candidate feature vector. Figure 1 depicts the workflow from an input record to window-level decisions and, for record-level tasks, the subsequent aggregation of decision weights. All fitted preprocessing and model-selection procedures are performed exclusively within the corresponding training fold.

### 2.2. Raw-Vibration View

Each window is centered and normalized by its root-mean-square (RMS) amplitude:

(4) $${\tilde{x}}^{\left(q\right)}{=x}^{\left(q\right)}-{\bar{x}}^{\left(q\right)},\;{\;x}_{r}^{\left(q\right)}=\frac{{\tilde{x}}^{\left(q\right)}}{\sqrt{L^{-1}\sum_{i=1}^{L}({\tilde{x}}_{i}^{\left(q\right)}{)}^{2}}}$$

If the RMS value does not exceed *ε* = 10^−12^, the corresponding raw-view sequence is set to a zero vector of the same length. The resulting raw view retains the normalized broadband vibration waveform.

### 2.3. Logarithmic Hilbert-Envelope View

The second view is derived from the same normalized raw-vibration window. The analytic signal and its amplitude envelope are defined as

(5) $$\;z^{\left(q\right)}{=x}_{r}^{\left(q\right)}+jH\left\{x_{r}^{\left(q\right)}\right\},\;{\;a}^{\left(q\right)}{=|z}^{\left(q\right)}|$$

The envelope is scaled by its median and compressed logarithmically:

(6) $$u^{\left(q\right)}=\log\left(1+\frac{a^{\left(q\right)}}{\mathrm{median}{(a}^{\left(q\right)})+\epsilon }\right)$$

The resulting series is RMS-normalized to obtain $x_{e}^{\left(q\right)}$. This procedure does not involve spectral-kurtosis-based band selection, label-guided filtering, or signal decomposition methods such as empirical mode decomposition. Accordingly, the envelope is used to characterize amplitude modulation rather than to isolate fault-induced impulses.

### 2.4. Multiscale Construction

For MPE and MFE, non-overlapping mean coarse graining is performed at each scale *s* in *S* = {1, 2, 3, 5, 8}:

(7) $$y_{j}^{\left(s\right)}=\frac{1}{s}\sum_{i=(j-1)s+1}^{\mathrm{js}}x_{i},\;j=1,\ldots ,\left\lfloor \frac{L}{s}\right\rfloor$$

For RCMFDE, at scale *s*, *s* coarse-grained sequences are constructed using the starting offsets *k* = 1,…,*s*. A pattern-probability vector is estimated for each valid sequence, and these vectors are averaged across offsets before the entropy is computed. This procedure differs from averaging entropy values computed separately from the individual coarse-grained sequences.

### 2.5. Multiscale Permutation Entropy

MPE characterizes the ordinal structure of a signal based on the permutation entropy framework [3]. For each coarse-grained sequence, embedding vectors are constructed with embedding dimension *m* = 3 and time delay *τ* = 1. Each embedding vector is mapped to an ordinal pattern *π* by stable sorting, with equal values ordered according to their original coordinate positions. Let *p*(*π*) denote the empirical probability of ordinal pattern *π*:

(8) MPEs=−∑πpπln pπlnm!

Five MPE values are obtained for each signal view, corresponding to the five prescribed scales. These values characterize scale-dependent ordinal dynamics without directly retaining amplitude information.

### 2.6. Multiscale Fuzzy Entropy

MFE characterizes local fuzzy similarity based on the continuous fuzzy-membership formulation of FuzzyEn [5]. At each scale, template vectors of dimensions *m* = 2 and *m* + 1 are constructed with time delay *τ* = 1, and the mean is removed from each template. The Chebyshev distance *d_ij_* between two templates is then transformed into a fuzzy similarity:

(9) $$\mu {(d}_{\mathrm{ij}})=\exp\left[-{\left(\frac{d_{\mathrm{ij}}}{r_{s}}\right)}^{n}\right],r_{s}=0.15\;\sigma {(y}^{\left(s\right)}),n=2$$

Self-matches are excluded from the similarity calculation. Let *ϕ^m^* and *ϕ^m^*^+1^ denote the aggregated non-self similarities for embedding dimensions *m* and *m* + 1, respectively:

(10) $$\mathrm{MFE}\left(s\right)=-\ln\left(\frac{\phi^{m+1}}{\phi^{m}}\right)$$

If the available templates or valid similarity values are insufficient for the calculation, MFE is treated as undefined rather than assigned a value of zero. These undefined values are retained and subsequently imputed using statistics estimated from the corresponding training fold.

### 2.7. Refined Composite Multiscale Fluctuation-Based Dispersion Entropy

RCMFDE characterizes amplitude-related fluctuations by combining fluctuation-based dispersion patterns [6] with refined composite multiscale probability aggregation [12]. Each signal view is mapped into *c* = 6 symbolic classes using the mean and standard deviation of the original view sequence. Symbolic embedding vectors are constructed with *m* = 2 and *τ* = 1, after which differences between adjacent embedded coordinates are calculated. The resulting difference alphabet contains *2c* − 1 = 11 possible fluctuation symbols. At each scale, a pattern-probability vector is estimated for each valid offset sequence, and these probability vectors are averaged across offsets before entropy calculation. Let ${\bar{p}}_{s}(\delta )$ denote the mean probability for fluctuation pattern *δ*:

(11) $$\mathrm{RCMFDE}\left(s\right)=-\frac{\sum_{\delta }{\bar{p}}_{s}\left(\delta \right)\ln{\bar{p}}_{s}\left(\delta \right)}{\ln\left[{\left(2c-1\right)}^{m-1}\right]}$$

Five RCMFDE values are obtained for each signal view, corresponding to the five prescribed scales. In contrast to MPE, RCMFDE retains information associated with amplitude classes; in contrast to MFE, it characterizes symbolic fluctuations rather than continuous template similarity.

### 2.8. Candidate Representation

The complete window-level representation is formed by concatenating the six five-dimensional entropy profiles:

**f**^(*q*)^ = [**f***_r_*_,MPE_, **f***_r_*_,MFE_, **f***_r_*_,RCMFDE_, **f***_e_*_,MPE_, **f***_e_*_,MFE_, **f***_e_*_,RCMFDE_] ∈ ℝ^30^ (12)

The representation varies along three design axes: signal view, entropy measure, and temporal scale. Its 30 dimensions are candidate features, not necessarily independent physical quantities or individually beneficial additions.

### 2.9. Training-Fold-Only Feature Control

All data-dependent preprocessing operations are fitted exclusively on the current outer training fold and then applied without refitting to the corresponding test fold:

(1) Undefined values are replaced by the corresponding feature median estimated from the training fold.

(2) Features with a training-fold variance less than or equal to 10^−14^ are removed.

(3) The remaining features are ranked according to their univariate ANOVA/Fisher scores.

(4) Features are examined in descending order of score. A lower-ranked feature is removed if its absolute correlation with any retained higher-ranked feature exceeds 0.95.

(5) The number of retained features, *K*, is selected from the task-specific candidate set by grouped inner cross-validation, subject to the number of available features after pruning.

(6) A Standard Scaler is fitted to the selected training features and then applied without refitting to the corresponding validation or test features.

If the training subset contains fewer than two inner groups for any class, the fixed fallback rule *K* = *min*(10, *D*) is used, where *D* denotes the number of features remaining after pruning.

In the original DCMEF source runs, the candidate feature counts were {6, 10, 14, 18} for SEU and HUST gearbox and {5, 10, 15, 20, 30} for UOttawa expanded and HUST bearing, each limited by the dimensions available after pruning. The view ablation followed the corresponding task configuration. A one-feature comparator cannot search the same numerical feature counts as a 30-feature bank; feasible candidate counts were limited by representation dimension.

### 2.10. Classifier Output and Record-Level Aggregation

DCMEF is not restricted to a specific classifier. In this study, it is evaluated with Extra Trees, Random Forest, and KNN. For a classifier providing decision scores *d_qk_*, a numerically stable softmax transformation is applied to obtain normalized decision weights:

(13) $$w_{\mathrm{qk}}=\frac{\exp{(d}_{\mathrm{qk}}-\underset{l}{\max}\;d_{\mathrm{ql}})}{\sum_{l}\exp{(d}_{\mathrm{ql}}-\underset{l}{\max}\;d_{\mathrm{ql}})}$$

For binary classification, a one-dimensional score is first written as [−*d_q_*,*d_q_*] before applying the softmax transformation. When predict_proba is available, its nonnegative outputs are renormalized to sum to one if necessary. Because no probability-calibration procedure is applied, the resulting quantities are interpreted as normalized decision weights rather than calibrated class probabilities.

For record-level evaluation, the decision weights are averaged across the *Q_r_* windows associated with record *r*:

(14) $${\bar{w}}_{\mathrm{rk}}=\frac{1}{Q_{r}}\sum_{q\in r}w_{\mathrm{qk}},{\hat{y}}_{r}=\arg\underset{k}{\max}{\bar{w}}_{\mathrm{rk}}$$

Thus, record-level prediction is based on the arithmetic mean of the window-level decision weights rather than majority voting. The SEU gearbox task is evaluated using window-level accuracy, whereas the other three tasks are evaluated using record-level accuracy. These two evaluation units are reported separately in Section 4.

## 3. Experimental Setup

### 3.1. Diagnostic Tasks

Four diagnostic tasks were included in this study. Table 1 summarizes the scope of each task and the grouping unit used for outer validation.

The SEU gearbox task used the 20-0 speed-load configuration of the source dataset and included five classes: Health, Chipped, Miss, Root, and Surface [28]. Channel 2 was partitioned into deterministic 8192-sample outer blocks, each yielding up to seven 2048-sample windows. The outer-validation split was defined at the block level, ensuring that windows originating from a held-out block were excluded from the corresponding training set. Accuracy for this task was computed from 140 pooled window-level predictions.

**Figure 2:**
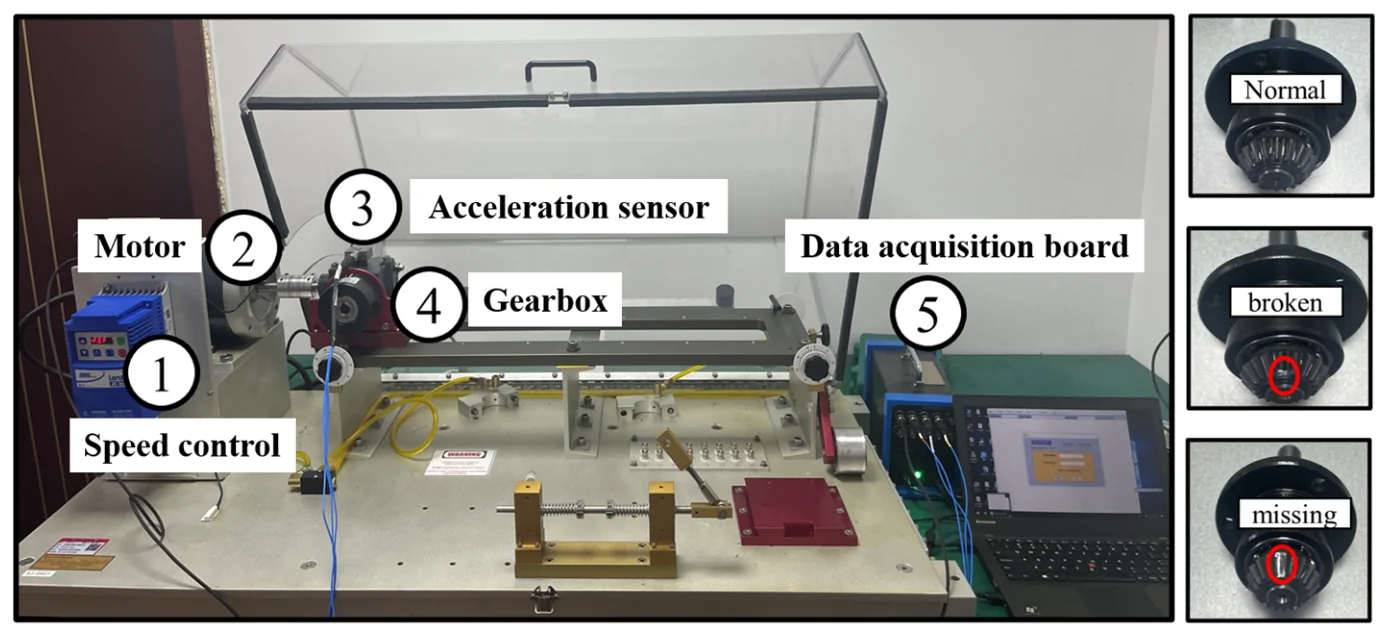
HUST gearbox test rig and fault gears.

The HUST gearbox task included three health states: healthy (H), broken-tooth (B), and missing-tooth (M) [29]. The x-axis vibration signal, sampled at 25.6 kHz, was used as the primary measurement channel. The dataset covered five steady-speed settings and five load conditions. Load condition was used as the grouping variable for outer validation, resulting in five leave-one-load-group-out folds. The analysis comprised 75 records and 900 windows, with window-level predictions aggregated at the record level before accuracy was computed (Figure 2).

**Figure 3:**
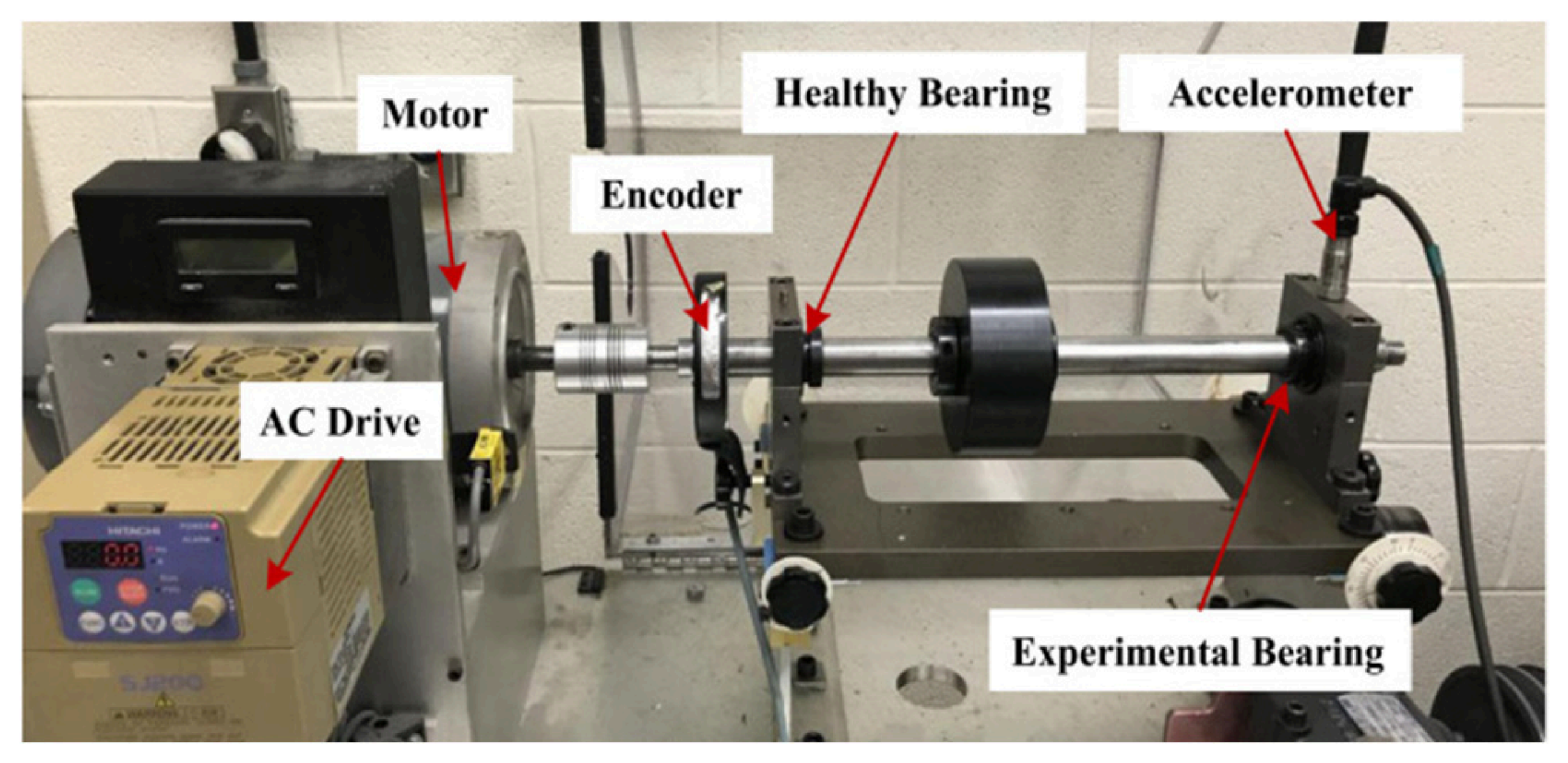
UOttawa variable-speed bearing test rig.

The expanded UOttawa task used Version 2 of the public variable-speed bearing dataset [30] and included five health states: healthy (H), inner-race fault (I), outer-race fault (O), ball fault (B), and combined fault (C). The vibration signals were sampled at 200 kHz. Four speed profiles were used as the outer-validation groups, with one profile held out in each outer fold. The task comprised 60 records and 720 windows. Accuracy was computed from pooled record-level predictions (Figure 3).

**Figure 4:**
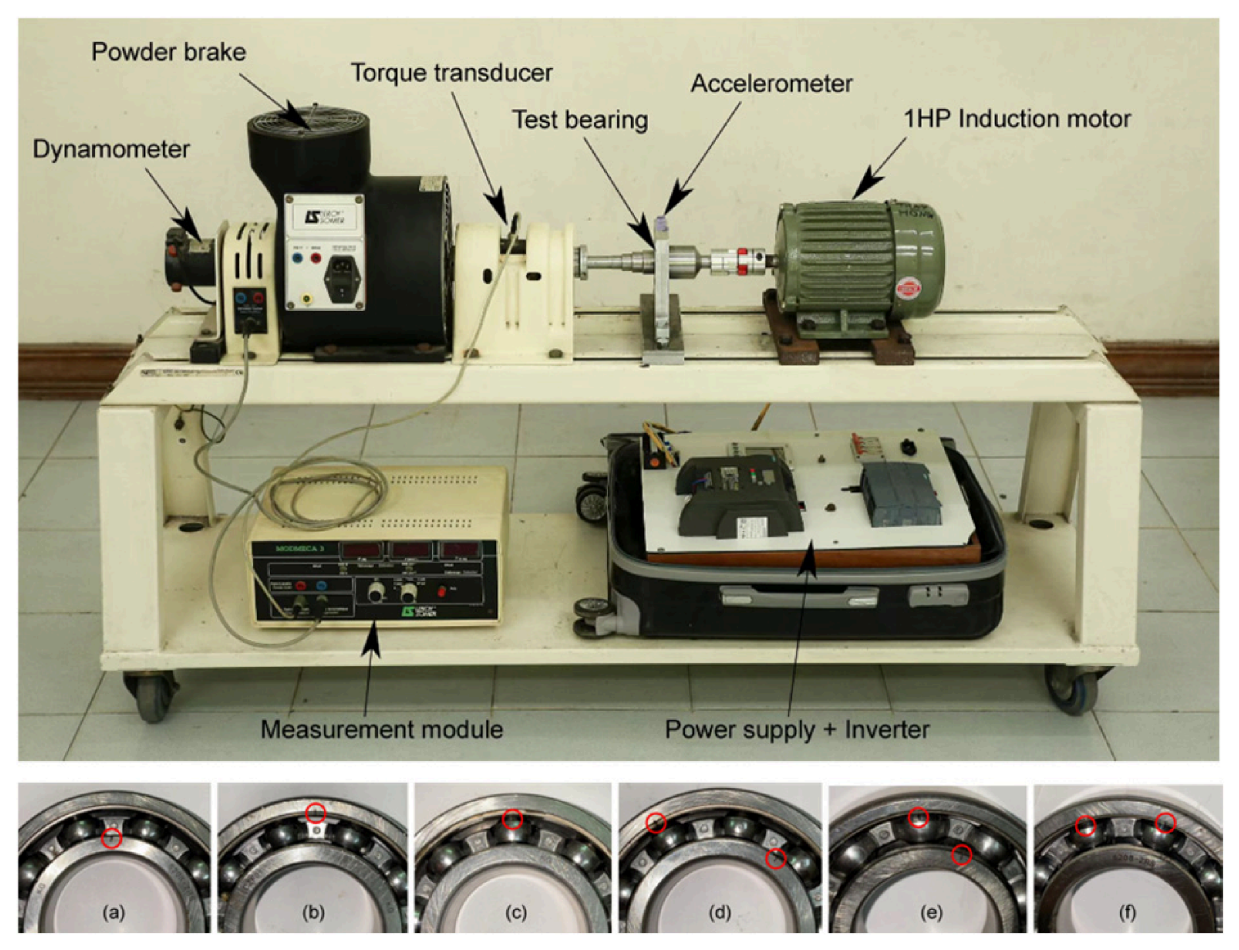
HUST bearing test rig and defects. (**a**) Inner-race; (**b**) outer-race; (**c**) ball; (**d**) inner- and outer-race; (**e**) inner-race and ball; and (**f**) outer-race and ball defects. Red circles mark artificial defect locations [31].

The HUST bearing task used 84 records from the public dataset [31]. It included seven health states: normal (N), inner-race fault (I), outer-race fault (O), ball fault (B), inner–outer fault (IO), inner–ball fault (IB), and outer–ball fault (OB), covering bearing models 6205–6208. Load conditions of 0, 200, and 400 W were used as the outer-validation groups. The task comprised 1008 windows, and record-level predictions were pooled across the three leave-one-load-group-out folds (Figure 4).

### 3.2. Compared Entropy Representations

The comparison comprised 21 feature representations. Five single-scale methods—PE, DE, SE, FE, and DivE—were included as the first group. Their standard multiscale counterparts were MPE, MDE, MSE, MFE, and MDivE. The composite multiscale variants comprised CMPE, CMDE, CMSE, CMFE, and CMDivE, while the refined composite multiscale variants comprised RCMPE, RCMDE, RCMSE, RCMFE, and RCMDivE. DCMEF constituted the twenty-first representation.

All multiscale comparator methods were evaluated at scales 1, 2, 3, 5, and 8. Their formulations followed the established single-scale, multiscale, composite multiscale, and refined composite multiscale frameworks [7,9,10,11,12]. The common parameter settings were *m* = 3 and *τ* = 1 for PE; *m* = 2, *c* = 6, and *τ* = 1 for DE; *m* = 2, *r* = 0.15 × SD, and *τ* = 1 for SE; *m* = 2, *r* = 0.15 × SD, fuzzy exponent 2, and *τ* = 1 for FE; and *m* = 4 with *ε* = 100 for DivE. Undefined entropy values were retained during feature extraction and subsequently imputed using statistics estimated exclusively from the corresponding outer training fold. For composite multiscale methods, entropy values were averaged across offset sequences. For refined composite multiscale methods, the relevant probability or similarity statistics were aggregated across offset sequences before the final entropy calculation. CMDivE and RCMDivE were implemented in this study for benchmarking the corresponding aggregation schemes and are not presented as methods proposed in the cited literature.

A 2048-sample window spans 80 ms at 25.6 kHz and 10.24 ms at 200 kHz. At scale 8, mean coarse-graining leaves 256 samples. For the DCMEF RCMFDE features, the embedding dimension is m = 2, and the number of symbolic classes is c = 6; m = 4 refers to the separate DivE comparator. Fixing the sample count and scales enables a common computational setting but does not give the same physical duration at different sampling rates or establish parameter optimality. The cached-feature audit found no undefined values across the 120 task-by-feature combinations inspected; this checks that values were computable, not that their sampling variance is small.

### 3.3. Classifiers and Nested Model Selection

Three classifiers were evaluated for each feature representation: Extra Trees, Random Forest, and KNN. The classifier set in the original 21-representation comparison was specified after the initial DCMEF results had been obtained, so its rankings are exploratory. The later view ablation reused each task’s original grouped outer and inner folds, classifier search, and feasible feature-count settings; its results are reported separately from the original comparison.

For the SEU and HUST gearbox tasks, the tree-based ensembles used 100 estimators, whereas 200 estimators were used for the UOttawa and HUST bearing tasks. For Extra Trees, the maximum depth was selected from {unrestricted, 10}, and the maximum number of features considered at each split was selected from {square root, all}. For Random Forest, the maximum depth was selected from {unrestricted, 10}. For KNN, the number of neighbors was selected from {3, 5, 9}, with either uniform or distance-based weighting and Minkowski order selected from {1, 2}. Applicable classifier hyperparameters and the number of retained features were selected by grouped inner cross-validation within each outer training split. The held-out outer group was used exclusively for final prediction. The analyses were implemented in Python 3.14.

### 3.4. Evaluation and Reporting

Accuracy was used as the primary evaluation metric. For *n* evaluation units,

(15) $$\mathrm{Accuracy}=\frac{1}{n}\sum_{i=1}^{n}I\left({\hat{y}}_{i}{=y}_{i}\right)$$

The evaluation unit was a window for SEU gearbox and a record for the other three tasks. At those units, the four tasks contained 28, 25, 12, and 12 examples per class, respectively. We compare representations only within a task and classifier and do not average accuracy across tasks. Table 2 and Table 3 report accuracy to two decimal places, while Table 4 also gives correct/total counts. Percentage-point gaps are calculated from the underlying counts before rounding; ties require equal correct counts under the same denominator.

## 4. Results

### 4.1. Complete Comparison

**Figure 5:**
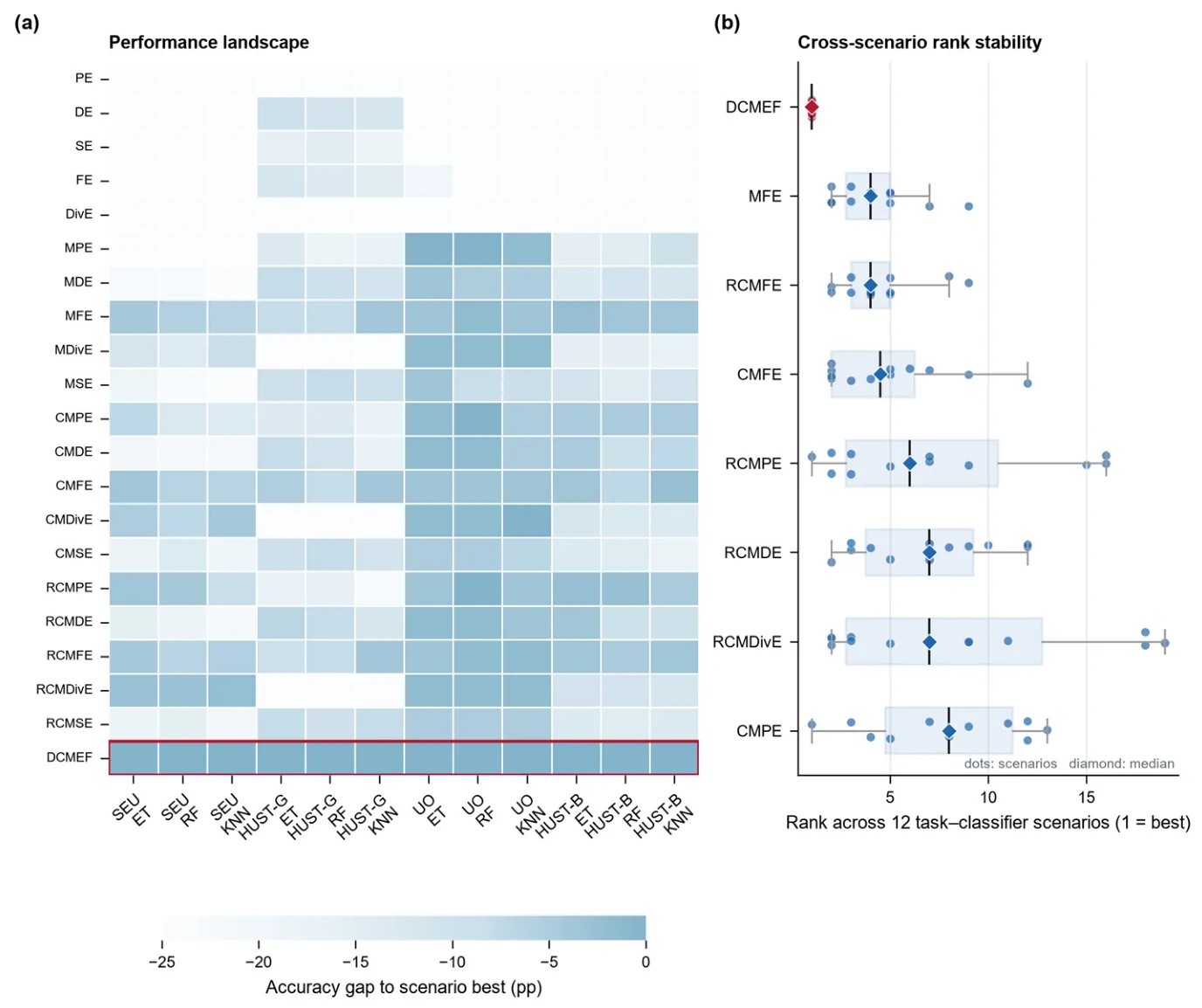
Accuracy gaps and rank stability across 12 task–classifier scenarios. (**a**) Accuracy gaps to the best result in each scenario; darker blue indicates smaller gaps, and the red outline marks DCMEF. The color scale is clipped at −25 percentage points. (**b**) Rank distributions: circles denote individual scenarios, diamonds denote median ranks, and boxes show interquartile ranges; red denotes DCMEF and blue denotes comparators.

**Figure 6:**
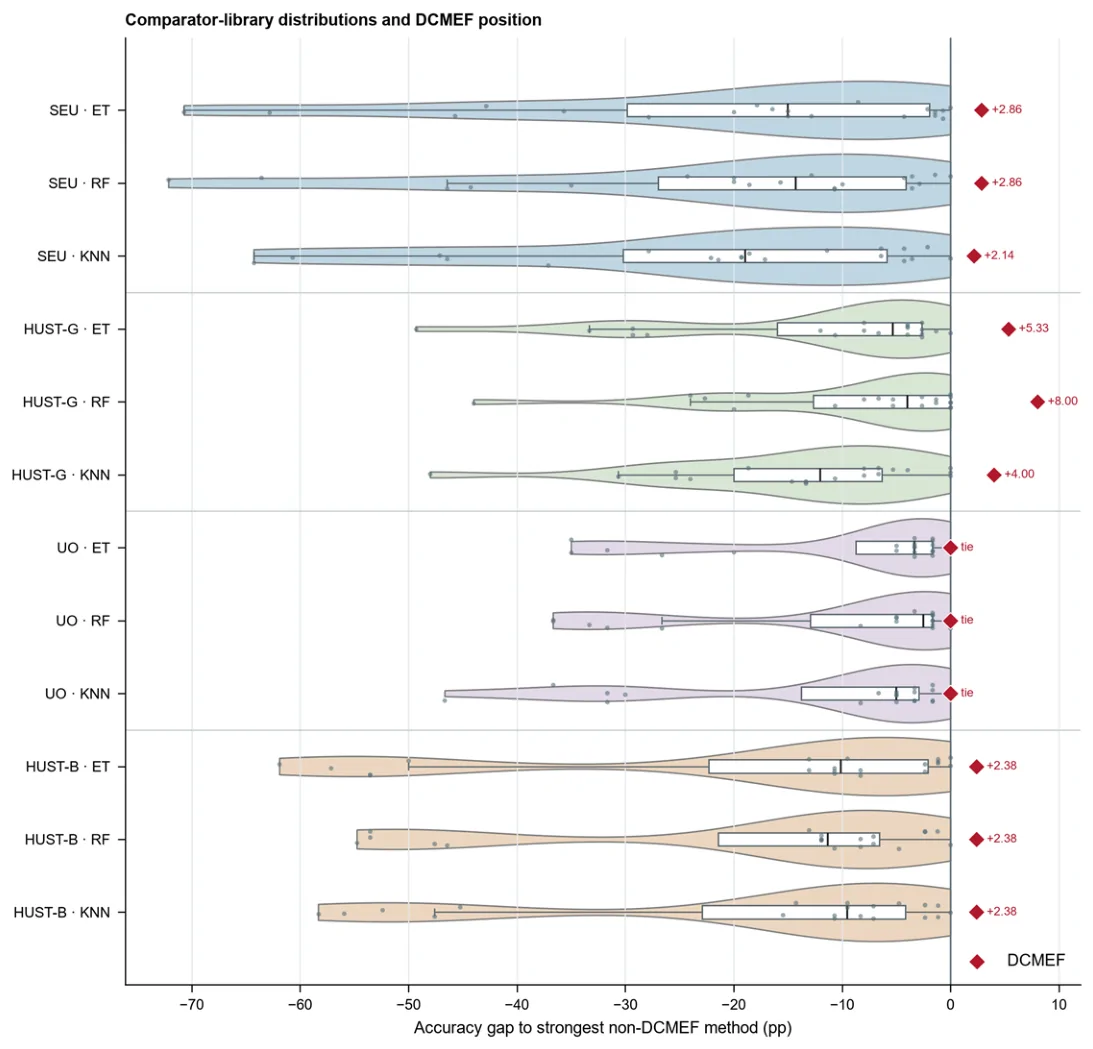
DCMEF margins relative to non-DCMEF comparator distributions. Blue, green, purple, and orange denote SEU gearbox, HUST gearbox, UOttawa, and HUST bearing. Shading shows comparator distributions; dots are comparator results and red diamonds are DCMEF. Zero denotes the strongest comparator.

Table 2 and Table 3 report the original 21-representation comparison across four tasks and three classifiers. Each column is interpreted at its task-specific reporting unit: windows for SEU gearbox and records for the other tasks. Boldface marks the highest observed accuracy, including exact ties. Table 4 reports correct/total counts and the strongest non-DCMEF comparator in each column. Figure 5 and Figure 6 summarize accuracy gaps and ranks within, rather than across, task–classifier settings.

In the original comparison, DCMEF had the highest observed accuracy in nine settings and tied for highest in the three UOttawa settings. The task and classifier matrix was assembled after earlier results had been seen, so these rankings describe the selected settings rather than independent confirmation.

### 4.2. Margins over the Strongest Non-DCMEF Representation

The nine positive gaps in Table 4 range from 2.14 to 8.00 percentage points; the three UOttawa gaps are zero. These gaps are differences between observed correct counts, not estimates of statistical significance.

### 4.3. Rank Stability Across Tasks and Classifiers

The strongest non-DCMEF representation varied by task and classifier. RCMDivE led the SEU comparators, fuzzy-entropy variants were strongest for HUST gearbox, and different entropy families led the remaining tasks. In the selected settings, DCMEF matched or exceeded these observed accuracies, but the changing comparator and post-result selection do not establish universal superiority.

**Figure 7:**
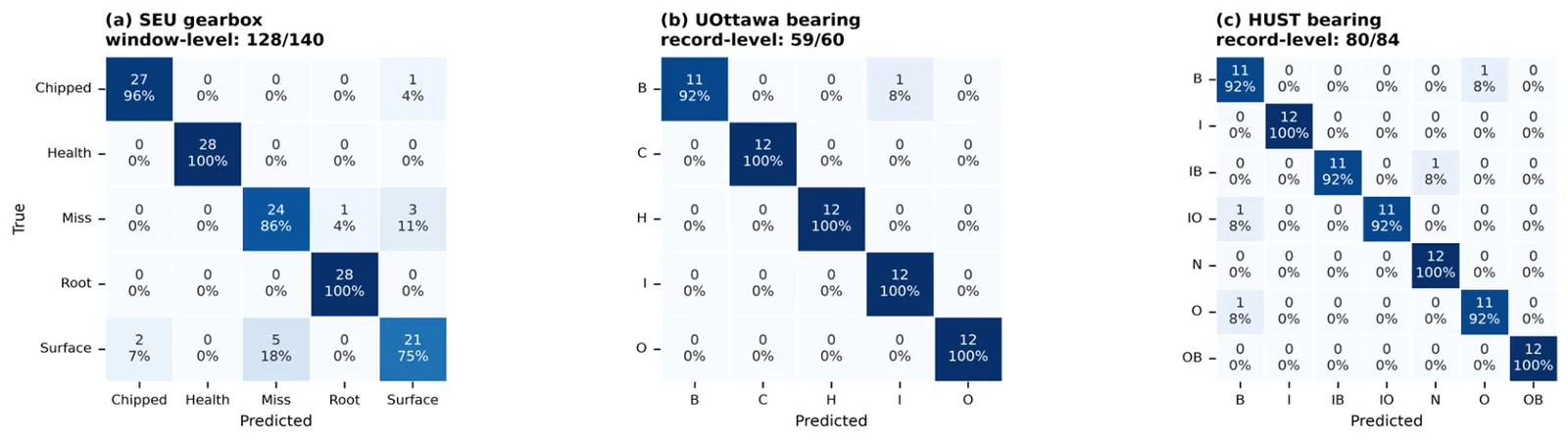
Extra Trees prediction-level confusion matrices for three tasks. (**a**) SEU gearbox; (**b**) UOttawa; (**c**) HUST bearing. Cells show counts and row percentages; darker blue indicates a higher percentage.

Figure 7 shows selected Extra Trees error patterns. In the UOttawa task, one ball-fault record was classified as an inner-race fault among 60 records. HUST bearing had four errors among 84 records, involving B–O, IB–N, IO–B, and O–B confusions. These matrices describe task-level error patterns; Figure 5 reports accuracy ranks across all four tasks and classifiers.

### 4.4. Summary of Results and View Ablation

In the original 21-representation comparison, DCMEF ranked first or joint first in each of the 12 within-task classifier settings. The three UOttawa ties show no additional accuracy gain. Because SEU is evaluated by windows and the other tasks by records, no accuracy is pooled across tasks.

SEU gearbox is evaluated by windows (*n* = 140); HUST gearbox, UOttawa expanded, and HUST bearing are evaluated by records (*n* = 75, 60, and 84). FULL results in this later rerun are not substituted into the original comparison in Table 2, Table 3 and Table 4.

FULL exceeded raw-only in two settings, tied in five, and was lower in five. It exceeded envelope-only in ten settings and tied in two. Raw-only exceeded FULL for SEU gearbox with KNN, all three HUST gearbox classifiers, and HUST bearing with KNN. The extra envelope features therefore helped in some settings but did not provide a consistent gain over raw-only features.

## 5. Discussion

### 5.1. Principal Finding

In the original comparison across the selected task–classifier settings, DCMEF ranked first without ties in nine settings and tied for first in three. The strongest comparator varied by setting, while the positive DCMEF gaps ranged from 2.14 to 8.00 percentage points. The separate view ablation qualifies that result: the full feature bank did not consistently outperform raw-only features.

All three UOttawa comparisons tied at 98.33%, so the full method preserved the highest observed accuracy there without an additional gain. The original comparison and later ablation were both performed on selected tasks after earlier results had been examined; their rankings are descriptive.

### 5.2. Interpretation of the DCMEF Representation

The two views describe different properties of the same vibration window: the raw waveform retains oscillatory structure, and the logarithmic Hilbert envelope emphasizes amplitude modulation. The view ablation tests their practical contribution without assuming that these properties always yield complementary classification gains. FULL exceeded envelope-only in ten settings but exceeded raw-only in only two; five raw-only settings performed better. Which entropy measure or scale is responsible for a change remains unresolved because those components were not removed separately.

**Figure 8:**
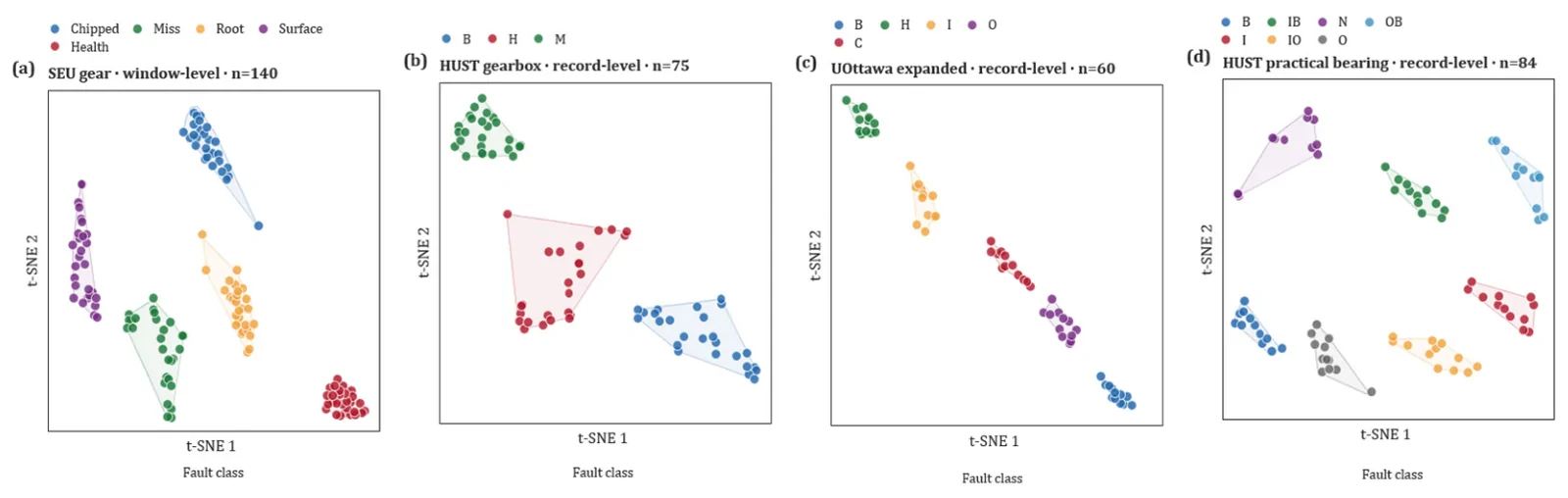
Exploratory t-SNE projections of the DCMEF feature bank. (**a**) SEU gearbox; (**b**) HUST gearbox; (**c**) UOttawa; (**d**) HUST bearing. Colors denote fault classes. Shading shows class-wise convex hulls, not confidence regions.

Figure 8 provides exploratory t-SNE projections of the DCMEF feature bank. Class overlap differs among the four tasks, but the projections do not measure predictive accuracy or isolate the effect of any feature group. Table 2, Table 3, Table 4 and Table 5 report the classification results and the direct signal-view contrasts.

### 5.3. Relationship to Previous Entropy-Based Diagnosis Studies

The identity of the strongest comparator varied across tasks and classifiers, which is consistent with the different coarse-graining strategies and estimation characteristics of multiscale entropy methods [9,18]. RCMDivE achieved the highest comparator accuracy for the SEU gearbox task. For the HUST gearbox task, the strongest alternatives were mainly fuzzy-entropy-based representations, whereas the HUST bearing task showed the best comparator results with either fuzzy-entropy or refined permutation-entropy variants, depending on the classifier. For UOttawa, the top-performing comparators included permutation- and diversity-entropy-based multiscale representations. These results indicate that the relative performance of an entropy family depends on the diagnostic task and operating condition and that results obtained from a single machine or operating regime are insufficient to identify a uniformly preferable entropy family.

DCMEF combines three entropy families and two signal views in a single candidate feature bank. In the original 21-representation comparison, DCMEF matched or exceeded the highest comparator accuracy in each task–classifier setting.

### 5.4. Practical and Methodological Implications

The original comparison placed DCMEF first or joint first with Extra Trees, Random Forest, and KNN. These classifiers partition features or use local feature-space distances in different ways, but the classifier set was fixed after earlier DCMEF results were examined. The observed agreement across classifiers is therefore exploratory; a prospective robustness test would pre-specify them before evaluating outcomes.

Measures to prevent data leakage are also important for interpreting the reported performance. Windows originating from the same physical record or operating group are not statistically independent; assigning related windows to both training and test sets could therefore inflate the estimated diagnostic performance. In the present workflow, outer groups were held out during evaluation, and all data-dependent transformations were fitted exclusively within the corresponding training folds. The evaluation units nevertheless differ across tasks: the SEU gearbox task reports pooled window-level accuracy, whereas the other three tasks report pooled record-level accuracy. Accordingly, Figure 5 and Figure 6 compare ranks and accuracy gaps only within individual task–classifier scenarios, and no mean accuracy is calculated across tasks.

### 5.5. Limitations

This study has several limits. The four-task, three-classifier comparison was assembled after earlier DCMEF results were seen, and the later view ablation used the same selected tasks; neither analysis is an independent confirmation. SEU accuracy is window-level, while the other tasks use records, so cross-task averages would mix different evaluation units. The positive gaps are descriptive because group-aware confidence intervals and paired tests were not established for the original comparison. UOttawa ties show no gain over the strongest comparator, and the view ablation shows that adding the envelope can lower accuracy. The separate effects of entropy families, scales, and feature-control stages remain unknown. The fixed 2048-sample window spans different physical durations across sampling rates; window and scale sensitivity, runtime, and selected-feature stability were not evaluated.

### 5.6. Confirmatory Evaluation

A confirmatory study should pre-specify the datasets, classifiers, primary metrics, independent groups, and analysis rules before outcomes are inspected. It should include external data and group-aware paired uncertainty estimates. Targeted removals of entropy families and scales, alternative window lengths, and runtime measurements would clarify the component effects and computational cost left open by the present analysis.

## 6. Conclusions

DCMEF combines RMS-normalized raw vibration with a normalized logarithmic Hilbert-envelope representation. Three entropy measures computed at five fixed scales yield 30 candidate features. Imputation, feature screening, feature selection, and standardization are performed using only data from the corresponding training folds.

In the original comparison on four selected tasks and three classifiers, DCMEF ranked first without ties in nine settings and tied for first in three; the positive gaps over the strongest comparator ranged from 2.14 to 8.00 percentage points. In a separate view ablation, FULL exceeded raw-only features in two of 12 settings, tied in five, and was lower in five, while it exceeded envelope-only in ten and tied in two. These results support the usefulness of the raw view and show that the value of adding the envelope depends on the setting. Because both analyses are exploratory, independent validation and targeted entropy, scale, and window studies are needed before broader performance or mechanism claims can be made.

## Figures and Tables

**Table 1:** Dataset and validation summary.

Task	Classes	Windows	Records/Outer Groups	Outer Protocol	Reported Accuracy Unit
SEU gearbox	5	140	20/20	Three-fold grouped split of deterministic 8192-point source blocks	Window-level pooled accuracy
HUST gearbox	3	900	75/5	Leave-one-load-group-out	Record-level pooled accuracy
UOttawa expanded	5	720	60/4	Leave-one-speed-profile-out	Record-level pooled accuracy
HUST bearing	7	1008	84/3	Leave-one-load-group-out	Record-level pooled accuracy

**Table 2:** Accuracy (%) on the SEU and HUST gearbox tasks.

Method	SEU ET	SEU RF	SEU KNN	HUST Gearbox ET	HUST Gearbox RF	HUST Gearbox KNN
PE	17.86%	17.14%	27.86%	54.67%	61.33%	60.00%
DE	45.71%	45.00%	42.14%	84.00%	82.67%	82.67%
SE	42.86%	42.86%	41.43%	77.33%	78.67%	76.00%
FE	52.86%	54.29%	51.43%	81.33%	80.00%	80.00%
DivE	25.71%	25.71%	24.29%	38.67%	41.33%	42.67%
MPE	60.71%	65.00%	60.71%	80.00%	74.67%	77.33%
MDE	68.57%	69.29%	67.14%	85.33%	84.00%	84.00%
MFE	87.14%	86.43%	84.29%	85.33%	85.33%	90.67%
MDivE	80.00%	78.57%	82.14%	58.67%	65.33%	66.67%
MSE	72.14%	69.29%	66.43%	84.00%	85.33%	85.33%
CMPE	84.29%	79.29%	77.14%	80.00%	80.00%	77.33%
CMDE	70.71%	70.71%	69.29%	85.33%	82.67%	77.33%
CMFE	87.86%	85.71%	84.29%	88.00%	85.33%	90.67%
CMDivE	86.43%	85.00%	86.43%	60.00%	66.67%	65.33%
CMSE	73.57%	78.57%	71.43%	84.00%	85.33%	84.00%
RCMPE	87.86%	87.86%	82.14%	76.00%	77.33%	72.00%
RCMDE	75.71%	73.57%	69.29%	86.67%	85.33%	82.67%
RCMFE	87.14%	85.71%	85.00%	84.00%	85.33%	90.67%
RCMDivE	88.57%	89.29%	88.57%	58.67%	62.67%	65.33%
RCMSE	73.57%	76.43%	70.00%	85.33%	84.00%	86.67%
DCMEF	**91.43%**	**92.14%**	**90.71%**	**93.33%**	**93.33%**	**94.67%**

Bold values indicate the highest observed accuracy within each column, including ties.

**Table 3:** Accuracy (%) on UOttawa and HUST bearing.

Method	UOttawa ET	UOttawa RF	UOttawa KNN	HUST Bearing ET	HUST Bearing RF	HUST Bearing KNN
PE	63.33%	61.67%	66.67%	42.86%	45.24%	45.24%
DE	71.67%	61.67%	68.33%	35.71%	44.05%	40.48%
SE	66.67%	71.67%	61.67%	30.95%	36.90%	36.90%
FE	78.33%	66.67%	66.67%	39.29%	38.10%	47.62%
DivE	63.33%	65.00%	51.67%	39.29%	38.10%	34.52%
MPE	**98.33%**	**98.33%**	96.67%	79.76%	79.76%	85.71%
MDE	95.00%	93.33%	93.33%	82.14%	83.33%	83.33%
MFE	95.00%	96.67%	95.00%	92.86%	90.48%	91.67%
MDivE	96.67%	96.67%	96.67%	79.76%	78.57%	78.57%
MSE	95.00%	90.00%	90.00%	84.52%	79.76%	84.52%
CMPE	96.67%	**98.33%**	93.33%	90.48%	89.29%	90.48%
CMDE	96.67%	96.67%	93.33%	90.48%	84.52%	88.10%
CMFE	95.00%	95.00%	95.00%	91.67%	86.90%	92.86%
CMDivE	96.67%	96.67%	**98.33%**	83.33%	80.95%	82.14%
CMSE	93.33%	93.33%	91.67%	82.14%	79.76%	77.38%
RCMPE	95.00%	**98.33%**	95.00%	92.86%	91.67%	90.48%
RCMDE	96.67%	96.67%	95.00%	91.67%	84.52%	85.71%
RCMFE	95.00%	96.67%	96.67%	91.67%	89.29%	91.67%
RCMDivE	96.67%	96.67%	96.67%	84.52%	83.33%	83.33%
RCMSE	93.33%	93.33%	93.33%	82.14%	79.76%	82.14%
DCMEF	**98.33%**	**98.33%**	**98.33%**	**95.24%**	**94.05%**	**95.24%**

Bold values indicate the highest observed accuracy within each column, including ties.

**Table 4:** Original-comparison correct counts, accuracy, and within-setting gaps for DCMEF and the strongest comparator.

Task	Classifier	DCMEF Correct/Total (Accuracy)	Comparator Correct/Total (Accuracy)	Gap (pp)	Outcome
SEU gearbox	Extra Trees	128/140 (91.43%)	124/140 (88.57%)	+2.86	Unique best
SEU gearbox	Random Forest	129/140 (92.14%)	125/140 (89.29%)	+2.86	Unique best
SEU gearbox	KNN	127/140 (90.71%)	124/140 (88.57%)	+2.14	Unique best
HUST gearbox	Extra Trees	70/75 (93.33%)	66/75 (88.00%)	+5.33	Unique best
HUST gearbox	Random Forest	70/75 (93.33%)	64/75 (85.33%)	+8.00	Unique best
HUST gearbox	KNN	71/75 (94.67%)	68/75 (90.67%)	+4.00	Unique best
UOttawa expanded	Extra Trees	59/60 (98.33%)	59/60 (98.33%)	0.00	Joint best
UOttawa expanded	Random Forest	59/60 (98.33%)	59/60 (98.33%)	0.00	Joint best
UOttawa expanded	KNN	59/60 (98.33%)	59/60 (98.33%)	0.00	Joint best
HUST bearing	Extra Trees	80/84 (95.24%)	78/84 (92.86%)	+2.38	Unique best
HUST bearing	Random Forest	79/84 (94.05%)	77/84 (91.67%)	+2.38	Unique best
HUST bearing	KNN	80/84 (95.24%)	78/84 (92.86%)	+2.38	Unique best

**Table 5:** Separate exploratory signal-view ablation (correct/total and accuracy).

Task	Classifier	FULL	Raw Only	Envelope Only
SEU gearbox	Extra Trees	128/140 (91.43%)	128/140 (91.43%)	85/140 (60.71%)
SEU gearbox	Random Forest	129/140 (92.14%)	119/140 (85.00%)	93/140 (66.43%)
SEU gearbox	KNN	124/140 (88.57%)	130/140 (92.86%)	84/140 (60.00%)
HUST gearbox	Extra Trees	65/75 (86.67%)	66/75 (88.00%)	60/75 (80.00%)
HUST gearbox	Random Forest	67/75 (89.33%)	69/75 (92.00%)	62/75 (82.67%)
HUST gearbox	KNN	66/75 (88.00%)	68/75 (90.67%)	61/75 (81.33%)
UOttawa expanded	Extra Trees	59/60 (98.33%)	59/60 (98.33%)	59/60 (98.33%)
UOttawa expanded	Random Forest	59/60 (98.33%)	59/60 (98.33%)	59/60 (98.33%)
UOttawa expanded	KNN	59/60 (98.33%)	59/60 (98.33%)	58/60 (96.67%)
HUST bearing	Extra Trees	80/84 (95.24%)	79/84 (94.05%)	62/84 (73.81%)
HUST bearing	Random Forest	77/84 (91.67%)	77/84 (91.67%)	63/84 (75.00%)
HUST bearing	KNN	78/84 (92.86%)	81/84 (96.43%)	66/84 (78.57%)

## Data Availability

The public datasets analyzed in this study are available from the sources cited in References [28,29,30,31]. Code, task configurations, and revision analysis outputs are retained by the authors and can be provided on reasonable request.

## References

[1] Liu L., Zhi Z., Zhang H., Guo Q., Peng Y., Liu D. (2019). Related Entropy Theories Application in Condition Monitoring of Rotating Machineries. Entropy.

[2] Randall R.B., Antoni J. (2011). Rolling Element Bearing Diagnostics—A Tutorial. Mech. Syst. Signal Process..

[3] Bandt C., Pompe B. (2002). Permutation Entropy: A Natural Complexity Measure for Time Series. Phys. Rev. Lett..

[4] Richman J.S., Moorman J.R. (2000). Physiological Time-Series Analysis Using Approximate Entropy and Sample Entropy. Am. J. Physiol. Heart Circ. Physiol..

[5] Chen W., Zhuang J., Yu W., Wang Z. (2009). Measuring Complexity Using FuzzyEn, ApEn, and SampEn. Med. Eng. Phys..

[6] Azami H., Escudero J. (2018). Amplitude- and Fluctuation-Based Dispersion Entropy. Entropy.

[7] Rostaghi M., Azami H. (2016). Dispersion Entropy: A Measure for Time-Series Analysis. IEEE Signal Process. Lett..

[8] Wang X., Si S., Li Y. (2021). Multiscale Diversity Entropy: A Novel Dynamical Measure for Fault Diagnosis of Rotating Machinery. IEEE Trans. Ind. Inform..

[9] Humeau-Heurtier A. (2015). The Multiscale Entropy Algorithm and Its Variants: A Review. Entropy.

[10] Costa M., Goldberger A.L., Peng C.-K. (2002). Multiscale Entropy Analysis of Complex Physiologic Time Series. Phys. Rev. Lett..

[11] Wu S.-D., Wu C.-W., Lin S.-G., Wang C.-C., Lee K.-Y. (2013). Time Series Analysis Using Composite Multiscale Entropy. Entropy.

[12] Azami H., Rostaghi M., Abásolo D., Escudero J. (2017). Refined Composite Multiscale Dispersion Entropy and Its Application to Biomedical Signals. IEEE Trans. Biomed. Eng..

[13] Li H., Huang J., Yang X., Luo J., Zhang L., Pang Y. (2020). Fault Diagnosis for Rotating Machinery Using Multiscale Permutation Entropy and Convolutional Neural Networks. Entropy.

[14] Gan X., Lu H., Yang G., Liu J. (2018). Rolling Bearing Diagnosis Based on Composite Multiscale Weighted Permutation Entropy. Entropy.

[15] Wu T.-Y., Yu C.-L., Liu D.-C. (2016). On Multi-Scale Entropy Analysis of Order-Tracking Measurement for Bearing Fault Diagnosis under Variable Speed. Entropy.

[16] Ying W., Tong J., Dong Z., Pan H., Liu Q., Zheng J. (2022). Composite Multivariate Multi-Scale Permutation Entropy and Laplacian Score Based Fault Diagnosis of Rolling Bearing. Entropy.

[17] Rostaghi M., Khatibi M.M., Ashory M.R., Azami H. (2021). Bearing Fault Diagnosis Using Refined Composite Generalized Multiscale Dispersion Entropy-Based Skewness and Variance and Multiclass FCM-ANFIS. Entropy.

[18] Wang X., Zhang Y., Wei Y., Ma C. (2026). A Review and Benchmark Study of Multi-Scale Entropy Methods for the Fault Diagnosis of Rotating Machinery. Entropy.

[19] Xu Y., He D., Luo X., Jin Z., Sun H. (2026). CSSCL: A cross-sensor self-contrastive learning for nondestructive fault detection of heavy locomotive motors in label-scarce scenarios. Nondestruct. Test. Eval..

[20] Xu Y., He D., Jin Z., Sun H., Zhang X., Zhang S., Fu Y. (2026). A fault diagnosis method for ship motors based on a multi-source data three-level fusion strategy. Ocean Eng..

[21] Liu B., Yan C., Liu Y., Lv M., Huang Y., Wu L. (2024). ISEANet: An interpretable subdomain enhanced adaptive network for unsupervised cross-domain fault diagnosis of rolling bearing. Adv. Eng. Inform..

[22] Liu B., Yan C., He C., Lv M., Wei J., Wu L. (2025). An interpretable physics-informed subdomain moment-enhanced adaptation network for unsupervised transfer fault diagnosis of rolling bearing. Adv. Eng. Inform..

[23] Shen Y., Shao H., Wang X., Zhang H., Xiao Y., Liu B. (2025). Compact-sparse prototype calibration network for few-shot continual fault diagnosis of rotating machinery. Eng. Appl. Artif. Intell..

[24] Liu L., Shao H., Xiao Y., Liu B. (2026). VAVFormer: A general interpretable fault diagnosis framework based on variational fusion and adaptive volatility attention. Comput. Ind..

[25] Wang J., Sun Z., Zhang J., Shao H., Xiao Y., Liu B. (2026). Towards generalizable fault diagnosis via LLM-driven hierarchical cross-modal alignment. Knowl.-Based Syst..

[26] Jin Z., Wu Z., He D., Zhao J., Fu Y. (2026). Data-driven nondestructive evaluation for bearing prognostics using a dual correlation graph knowledge distillation network. Nondestruct. Test. Eval..

[27] Jin Z., Gao Y., He D., Zhao J., Wu Z., Huang C.-G. (2026). A Multiscale Method for Predicting the Remaining Useful Life of Bearings Using Physics-Informed Hypergraph Wavelet Attention Network. IEEE Trans. Reliab..

[28] Shao S., McAleer S., Yan R., Baldi P. (2019). Highly Accurate Machine Fault Diagnosis Using Deep Transfer Learning. IEEE Trans. Ind. Inform..

[29] Zhao C., Zio E., Shen W. (2024). Domain Generalization for Cross-Domain Fault Diagnosis: An Application-Oriented Perspective and a Benchmark Study. Reliab. Eng. Syst. Saf..

[30] Huang H., Baddour N. (2019). Bearing Vibration Data Under Time-Varying Rotational Speed Conditions, Version 2.

[31] Thuan N.D., Hong H.S. (2023). HUST bearing: A practical dataset for ball bearing fault diagnosis. BMC Res. Notes.

